# Cellular senescence in age-related cardiovascular disease: past and future

**DOI:** 10.3389/fragi.2025.1721744

**Published:** 2026-01-12

**Authors:** Xiang Wu, Qingyu Zhou, Yingying Huang, Wangqing Jiang, Jianming Zhou, Ke Qian, Yinchen Pan, Zuyao Wu, Jingjun Zhang, Madinai Aimaiti, Qi Zhou, Feizhou Lv, Yong Lin, Shaomin Li, Shuying Chen

**Affiliations:** 1 Department of Laboratory Medicine, Huashan Hospital, Fudan University, Shanghai, China; 2 Shanghai Medical College, Fudan University, Shanghai, China; 3 Department of Clinical Laboratory, Central Laboratory, Jing’an District Center Hospital of Shanghai, Fudan University, Shanghai, China; 4 Department of Rehabilitation Medicine, The Sixth People’s Hospital Affiliated to Shanghai Jiao Tong University School of Medicine, Shanghai, China; 5 Department of Spine Surgery, Huashan Hospital, Fudan University, Shanghai, China; 6 Department of Laboratory Medicine, Shanghai Fifth People’s Hospital, Fudan University, Shanghai, China; 7 Ann Romney Center for Neurologic Diseases, Department of Neurology, Brigham and Women’s Hospital and Harvard Medical School, Boston, MA, United States

**Keywords:** age-related, cardiomyocytes, cells, cellular, CVD, DDR, endothelial, senescence

## Abstract

Cellular senescence is a distinct and definable biological state characterized by irreversible cell cycle arrest, accompanied by the activation of the DNA damage response (DDR), telomere shortening, the senescence-associated secretory phenotype (SASP), and metabolic dysfunction. While senescent cells represent only a small fraction of the total cell population in tissues, they exert a disproportionate and systemic impact on age-related cardiovascular disease (CVD) through paracrine and endocrine mechanisms. This review moves beyond a descriptive list of pathways and instead proposes a unified framework centered on how a small number of senescent cells can reprogram the cardiovascular microenvironment. We focus on the SASP as the central executor of this systemic effect, disseminating local senescence and driving chronic inflammation, fibrosis, and dysfunction across major cardiovascular cell types (cardiomyocytes, endothelial cells, fibroblasts, smooth muscle cells). We integrate key regulatory networks such as mTOR, AMPK, and Sirtuins that modulate the SASP and the senescent state. Furthermore, we discuss the translational promise of senolytics (agents that clear senescent cells) and senomorphics (agents that suppress the SASP) as novel strategies for delaying cardiovascular aging and treating age-related CVD, providing a forward-looking perspective on targeting senescence to promote cardiovascular health. Current research challenges include mechanistic complexity and limitations of animal models and *in vitro* systems. In the future, it is necessary to combine single-cell sequencing, metabolic intervention, and interdisciplinary technologies to analyze the heterogeneity of cellular aging, and develop early warning and precision treatment strategies based on aging biomarkers, so as to provide new ideas for delaying cardiovascular aging.

## 1 Introduction

Cellular senescence is a permanent state of cell cycle arrest characterized by pronounced metabolic activity and dramatic changes in cell morphology. Cardiovascular disease (CVD) refers to a class of diseases involving the heart and vascular system, mainly including coronary heart disease, hypertension, heart failure, arrhythmia, atherosclerosis, stroke, etc. Aging has been recognized as an independent risk factor for the development of cardiovascular disease (Jl et al., 2015). A growing body of clinical and laboratory evidence suggests that even in individuals without explicit CVD, the aging process promotes the structural and functional remodeling of the heart. There is some debate as to whether these aging-related changes are a phenotype of CVD, but it is undeniable that these changes do make the cardiovascular system more susceptible to CVD.

It has been confirmed that aging leads to structural changes in the heart, and compared with young individuals, aging hearts have macrostructural changes such as increased epicardial adipose tissue deposition, calcification in some areas, atrial remodeling, and decreased left ventricular systolic and diastolic volumes (Keller and Howlett, 2016). These structural changes can lead to impaired function, which in turn can lead to disease. For example, adipose tissue deposits may increase the risk of atrial fibrillation (Florio et al., 2020). Calcification of the aortic valve leaflets affects its function, impairing left ventricular ejection and potentially causing heart failure (Wang and Bennett, 2012). It can be seen that aging can lead to more CVD in the elderly by changing cardiovascular function. Here we give the definition of age-related cardiovascular disease, that is, cardiovascular diseases or changes in the cardiovascular system that are closely related to aging. It mainly includes atherosclerosis, heart failure, high blood pressure, arrhythmia, and so on (Florio et al., 2020; Wang and Bennett, 2012; Robles and Macias, 2015; Zhou et al., 2023).

The specific mechanism of age-related cardiovascular disease has not been fully elucidated, but it is related to cellular senescence. Cellular senescence is an important marker of aging and a key factor affecting the repair and regeneration of damaged cells in cardiovascular tissue (Childs et al., 2018). Cellular senescence is a stress-response process activated by various stressors, including reactive oxygen species, pro-inflammatory cytokines, metabolic, mechanical, and chemical toxicities such as sugar, fat, and chemical toxicity.

As the correlation between cellular senescence and the occurrence of age-related CVD is increasingly confirmed, it is important to study the mechanisms behind it to facilitate drug development and disease intervention. A critical paradox is that senescent cells, which constitute only a minor fraction of cells in aged tissues, can orchestrate tissue-wide dysfunction. This review poses and addresses the central question: how does a small population of senescent cells systemically drive cardiovascular pathogenesis? The answer lies in the SASP, which transforms senescence from a cell-intrinsic anti-cancer mechanism into a potent mediator of chronic tissue remodeling and dysfunction. This review will focus on the above aspects and introduce the relevant research progress in recent years.

## 2 Mechanisms of cell senescence and SASP secretion

### 2.1 Mechanisms of cellular senescence

**FIGURE 1:**
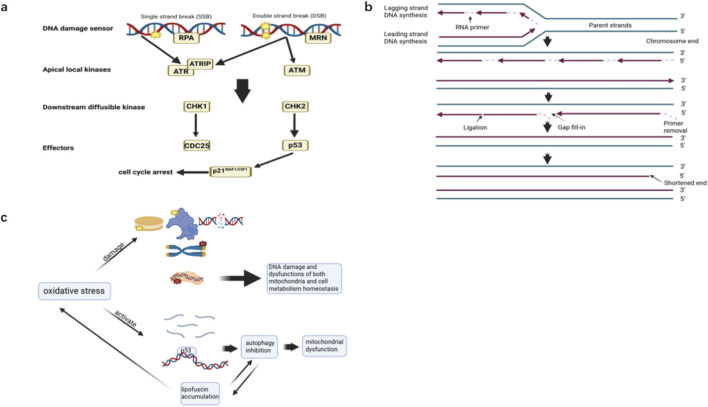
Mechanisms of Cellular Senescence. **(a)** The classic DDR pathway. When DNA damage occurs, there are various different DNA damage sensors that detect the damage, such as Replication Protein A (RPA) detecting exposed single-strand breaks and the MRE11-RAD50-NBS1 (MRN) complex detecting double-strand DNA breaks. They recruit upstream protein kinases ataxia telangiectasia mutated (ATM) and ataxia-telangiectasia and RAD3-related (ATR) to the damage site. Although both ATM and ATR are activated upon DNA damage, they have different DNA specificities; ATM is primarily activated by Double-strand breaks (DSB), while ATR responds to a wide range of DNA damage in addition to double-strand breaks. CHK1 and CHK2 are downstream diffusible kinases that function distally to the DNA damage, propagating the damage signal by phosphorylating final effector substrates such as p53. CHK1 negatively regulates CDC25 by phosphorylating Serine-216, CDC25 being a dual-specificity phosphatase that promotes the transition from G2 phase to M phase, resulting in G2 phase growth arrest. Phosphorylation of the Serine-20 site on p53 by CHK2 leads to a reduced affinity of the E3 ubiquitin ligase MDM2 for p53, thereby increasing the level of p53. The phosphorylated p53 upregulates the expression of p21WAF1/CIP1, a potent general CDKI, resulting in cell cycle arrest. **(b)** End replication problem. During the lagging strand DNA replication process, the RNA primer at the extreme 5′ end is removed, leaving a gap that cannot be filled by DNA polymerase. This results in the loss of DNA at chromosome ends during each replication cycle. **(c)** Oxidative stress induces cellular senescence. Excessive accumulation of ROS can damage biomolecules such as DNA, proteins, and lipids within cells. Oxidative stress damage from ROS also leads to faster telomere shortening. In addition, oxidative stress leads to mtDNA damage, induction of miR-210 and miR-494, and activation of p53 that mediates induction of autophagy inhibition. Mitochondrial dysfunction leads to cellular senescence due to mechanisms such as enhanced oxidative stress and activation of inflammatory signals. The two interact with each other and work together to promote cellular senescence.

The mechanisms of cellular senescence are complex and diverse, involving a variety of intracellular signaling pathways, metabolic processes and cell-to-cell interactions, and the following three mechanisms are introduced: DNA damage, telomere shortening and telomerase regulation, oxidative stress and mitochondrial dysfunction. As shown in Figure 1, these three mechanisms interact with each other and work together to promote cellular senescence.

#### 2.1.1 DNA damage

DNA damage such as single-strand and double-strand breaks can activate the DNA damage response (DDR), and classical DDR mainly involves the p53/p21WAF1/CIP1 pathway. As summarized in Figure 1, DNA damage activates ATM/ATR kinases, which in turn phosphorylate downstream targets (such as p53, CHK2), inducing cell cycle arrest. Cells that remain in arrest for an extended period may enter a state of senescence (Kumari and Jat, 2021; d'Adda di Fagagna, 2008). Prominent short-term DNA damage activates apoptosis, while long-term mild DNA damage induces cellular senescence (Petrova et al., 2016). In addition, high levels of nuclear DNA damage can disrupt mitochondrial function and mitophagy (Fang et al., 2014; Schumacher et al., 2021), while mitochondrial dysfunction can lead to cellular senescence.

#### 2.1.2 Telomere shortening and telomerase regulation

Telomere shortening has multiple causes, primarily the “end replication problem” as summarized in Figure 1 (Soudet et al., 2014); In addition, the formation of a hybrid DNA/RNA structure that interferes with replication by TERRA RNA (Telomeric Repeat-Contain RNA) also results in the loss of telomere sequences during replication (Metcalfe and Olsson, 2022); Furthermore, ROS contributes to telomere shortening: ROS can cause single-stranded DNA or double-stranded DNA breaks (SSBs or DSBs), which can affect telomere sequence integrity and cause telomere damage; In addition, it can affect telomere repair by impairing telomerase activity, resulting in telomere shortening (Ah et al., 2018). Oxidative stress damage from ROS also leads to faster telomere shortening (Metcalfe and Olsson, 2022). When the shortened telomere reaches a critical length, it is recognized as a DNA double-strand break and initiates the DDR process, resulting in a long period of cell cycle arrest in the G1 phase (Zhu et al., 2019).

#### 2.1.3 Oxidative stress and mitochondrial dysfunction

Oxidative stress refers to the phenomenon that the level of ROS in cells increases dramatically and causes damage to cells. As summarized in Figure 1, excessive accumulation of ROS can damage biomolecules such as DNA, proteins, and lipids within cells (Zhang L. et al., 2024; Ding et al., 2021; Liu et al., 2022; Wang et al., 2019). Oxidative stress damage from ROS also leads to faster telomere shortening (Erusalimsky, 2020). In addition, oxidative stress leads to mtDNA (Mitochondrial DNA, which is a unique class of genetic material found in mitochondria) damage, induction of miR-210 and miR-494, and activation of p53 that mediates induction of autophagy inhibition, all of which have been implicated in cellular senescence (Davalli et al., 2016). Mitochondrial dysfunction leads to cellular senescence due to mechanisms such as enhanced oxidative stress and activation of inflammatory signals (Harrington et al., 2023; Victorelli et al., 2023). The two interact with each other and work together to promote cellular senescence.

### 2.2 Mechanisms of SASP secretion

Senescent cells secrete a large number of factors, including pro-inflammatory cytokines, chemokines, growth regulators, angiogenesis factor, and matrix metalloproteinases (MMPs) and so on, which is known as the senescence-associated secretory phenotype (SASP) or senescence information secretome. SASP is a major feature of senescent cells and is involved in the regulation of related pathophysiological mechanisms. For example, SASP strengthens and spreads aging in an autocrine and paracrine manner, and also leads to long-term chronic inflammation. The composition and intensity of SASPs vary greatly depending on the duration of aging, the source of pro-aging stimuli, and cell type (Childs et al., 2015; Birch and Gil, 2020). For example, single-cell sequencing confirmed cell-to-cell differences in SASP secretion and expression (Wiley et al., 2017). Dysfunctional mitochondria can trigger a distinct form of senescence, known as mitochondrial dysfunction-associated senescence (MiDAS), which is devoid of the IL-1-dependent pro-inflammatory response typically seen in the SASP (Wiley et al., 2016). During oncogene-induced senescence (OIS), fluctuations in NOTCH1 levels drive the transition of the secretome from an early, TGF-β-rich, immunosuppressive state to a proinflammatory SASP (Hoare et al., 2016). Furthermore, in late-stage replicative senescence, stress-induced senescence, and OIS cells, the expression of type I interferons (IFN-I) emerges as a defining feature of the SASP (De et al., 2019).

#### 2.2.1 DNA damage response as a central driver of SASP activation

Senescent cells activate a robust DNA damage response (DDR), first recognized by d’Adda di Fagagna et al., in 2003, who showed that senescence is accompanied by persistent DNA damage foci marked by γH2AX and 53BP1 (d'Adda di Fagagna et al., 2003). Several years later, Rodier et al. demonstrated that this persistent DDR, rather than transient DNA breaks, drives chronic inflammatory signaling and SASP production (Rodier et al., 2009). Although canonical DDR kinases such as ATM and CHK2 initiate senescence-associated growth arrest, emerging work has revealed that SASP maturation requires a delayed, non-canonical DDR. This pathway involves the accumulation of ATM and the MRN complex on chromatin, which enables NF-κB recruitment without requiring ATM kinase activity (Malaquin et al., 2020). Related research finds that inhibiting NF-κB can delay the aging phenotypes associated with DNA damage, supporting the function of NF-κB in SASP induction (Tilstra et al., 2012).

#### 2.2.2 Mitochondrial dysfunction and cytosolic DNA sensing as drivers of SASP activation

The link between cGAS–STING signaling and senescence was first established by Glück et al., in 2017, who demonstrated that cytosolic chromatin fragments (CCF) activate cGAS to reinforce senescence (Glück et al., 2017). Later that same year, Dou and colleagues further showed that these chromatin fragments drive SASP production through cGAS–STING activation (Dou et al., 2017). The specific mechanism is CCF act as a ligand of the DNA sensor, cGAS-STING. The cGAS, cyclic GMP-AMP synthase, triggers the reaction to produce cyclic di-nucleotide, cyclic GMP-AMP (cGAMP), that is recognized by STING, thereby facilitating the type 1 interferon-producing pathway activated by phosphorylated IRF3 (Ohtani, 2022). However, the upstream mechanism of CCF formation was not clear at that time. In 2019, Maria Grazia Vizioli and colleagues found that dysfunctional mitochondria activate a ROS–JNK–mediated retrograde signaling pathway that promotes the formation of cytoplasmic chromatin fragments (CCFs), thereby triggering the SASP (Vizioli et al., 2020). This finding suggests that CCF generation is closely linked to mitochondrial dysfunction. Zhi-hua Zheng, Jiao-jiao Wang, and colleagues demonstrated that mtDNA-driven activation of the STING signaling pathway promotes vascular endothelial cell senescence and disrupts mitochondrial homeostasis. The authors also identified cilofibrate as a novel STING inhibitor capable of mitigating vascular endothelial inflammation and aging (Zheng et al., 2024). At the same time, related research further indicates that the production of mtDNA is related to aging. Minatory MOMP (miMOMP), which means mitochondrial outer membrane permeabilization (MOMP) occurring in a small subset of mitochondria without inducing cell-death, is a feature of senescent cells (Victorelli et al., 2023). In 2023, Stella Victorelli et al. reported that miMOMP, mediated by BAX- and BAK-formed macropores, leads to the release of mitochondrial DNA (mtDNA) into the cytosol, which subsequently activates the cGAS–STING signaling pathway and triggers SASP production (Victorelli et al., 2023). In addition to miMOMP, recent studies indicate that nucleotide metabolism imbalance can also lead to the release of mtDNA into the cytoplasm. Amir Bahat and Dusanka Milenkovic et al. found that imbalanced nucleotide synthesis triggers the release of mitochondrial DNA (mtDNA) into the cytoplasm and induces an innate immune response via the cGAS-STING signaling pathway. Their further research revealed that the cause of this mtDNA leakage is the incorrect incorporation of ribonucleotides during synthesis, and the addition of exogenous deoxyribonucleic acid can suppress this inflammatory response (Bahat et al., 2025).

Together, these findings indicate that aging promotes SASP secretion through multiple stress-associated pathways—including persistent DDR, mitochondrial dysfunction, and cytosolic DNA sensing—which converge on NF-κB and cGAS–STING to drive inflammatory gene expression.

## 3 The ways and types of SASP promoting the aging of organism

SASP plays a central role in amplifying senescence signals by converting cells’ autonomous aging programs into tissue dysfunction, thereby driving the emergence of systemic aging phenotypes.

### 3.1 SASP-mediated paracrine senescence

Paracrine senescence is the primary mechanism driving systemic aging. Members of the TGF-β family, vascular endothelial growth factor (VEGF), and chemokines CCL2 and CCL20 can transmit senescence to neighboring cells through inflammatory microsomes and IL-1 signaling, a process termed paracrine senescence (Acosta et al., 2013). Studies demonstrate that senescent cells induce DNA damage, telomere impairment, and aging in healthy adjacent cells via secreted senescence-associated secretory proteins (SASP) (Nelson et al., 2012; Victorelli et al., 2019). For instance, transplanting aged bone marrow cells or injecting senescent bone marrow models into young mice accelerates aging in the recipients (Hou et al., 2024). Transplanting a small number of pre-senescence adipocytes into young mice with minimal endogenous aging triggers persistent dysfunction of the victory function (Xu et al., 2018). Senescent melanocytes induce paracrine telomere damage in peripheral cells and affect epidermal renewal by inhibiting basal keratinocyte proliferation (Victorelli et al., 2019).

### 3.2 SASP-mediated autocrine senescence

The classical SASP autocrine senescence refers to a cell-autonomous process where senescent cells secrete SASP, which directly interacts with their surface receptors to enhance and sustain their senescent state over time (Volkó et al., 2019). In the non-classical autocrine senescence mechanism, the key SASP factor IL-6 maintains and enhances its interaction with intracellular IL-6R in the cis-traffic structure through cytoplasmic DNA and cGAS-STING-NFκB activation, thereby sustaining cellular autonomous senescence (Herbstein et al., 2024). Studies have shown that in the pituitary gland, IL-6 induces the proliferation of pituitary tumor cells through paracrine signaling. Subsequently, these tumor cells produce IL-6 during the tumor phase, triggering autocrine senescence and leading to invasive growth and malignant development (Herbstein et al., 2024; Sapochnik et al., 2017).

### 3.3 SASP promotes systemic aging via endocrine-like mechanisms

Senescent cells disseminate SASP through the circulatory system, disrupting homeostasis in distant tissues, inducing inflammation, and accelerating aging. By secreting pro-inflammatory factors (e.g., IL-1β and IL-6) from SASP into the bloodstream, these cells establish a chronic, low-grade systemic inflammatory state (Clayton et al., 2023). Studies demonstrate a strong correlation between chronic systemic inflammation and aging-related phenotypes in the elderly, including mortality risk and cognitive decline (Arai et al., 2015). It has been confirmed that this SASP-driven systemic inflammation serves as a common pathological basis for multiple age-related diseases (Giunta et al., 2022). In these inflammatory conditions, levels of SASP-associated pro-inflammatory cytokines and other mediators progressively rise in the bloodstream, observed in disorders such as Parkinson’s disease, Alzheimer’s disease, atherosclerosis, myocarditis, fatty liver, type 2 diabetes, osteoporosis, and cancer (Freund et al., 2010).

### 3.4 Prospects in the cardiovascular context

The paracrine, autocrine, and endocrine mechanisms of SASP dissemination provide a framework for understanding how localized senescence escalates into systemic cardiovascular aging. Paracrine senescence likely dominates in age-related CVD, where senescent cells recruit and corrupt neighbors. For instance, in pulmonary hypertension, endothelial cell senescence appears to induce dysfunction in other vascular cells by upregulating multiple canonical SASP factors (e.g., IL-6, TNF, and PAI-1) (van der Feen et al., 2020; Culley et al., 2021). Vascular endothelial cell senescence promotes the formation of inflammatory and thrombotic microenvironments through the secretion of SASP, leading to the degeneration of vascular wall structure and function, thereby facilitating the development and progression of CVD (Bu et al., 2023). Senescent cardiomyocytes undergo SASP, which upregulated senescence-related secretory factors, including CCN family member 1 (CCN1), IL-1α, TNF-α, TGF-β, and monocyte chemoattractant protein, which can induce dysfunction and senescence of endothelial cells (Cui et al., 2018; Tang et al., 2020). SASP-mediated autocrine senescence may be crucial in sustaining the senescent state of endothelial cells under chronic shear stress (Jia et al., 2022). Most importantly, senescent cells release SASP through the circulatory system, disrupting homeostasis in distal tissues, inducing inflammatory responses, and accelerating the aging process. This may provide a direct mechanistic link between focal cellular senescence in organs such as adipose tissue or the liver and the development of cardiovascular pathologies, including systemic endothelial dysfunction and arterial stiffness.

## 4 The role of cellular senescence in age-related cardiovascular disease

Building upon the definition of age-related CVD provided in the Introduction, this section delves into the central role of cellular senescence in driving the pathogenesis of these conditions. As shown in Table 1, the cell types associated with age-related cardiovascular disease include: cardiomyocytes, endothelial cells, fibroblasts, smooth muscle cells, etc. Given the pivotal role of cardiomyocytes and endothelial cells in the cardiovascular system, we concentrate on these two cell types.

**TABLE 1:** The relevant cell types and their mechanisms leading to age-related CVD.

Cell type	Mechanisms of cellular senescence leading to age-related CVD	References
Cardiomyocyte	Impaired contractility and abnormal conduction patterns of senescent cardiomyocytes	Gude et al. (2018), Chadda et al. (2018), Chen et al. (2022), He et al. (2024)
	May increase the risk of ventricular arrhythmia	
	By activating inflammatory signaling pathways and releasing pro-inflammatory cytokines, a chronic inflammatory state is maintained	
	By secreting SAS p factor, it influences the physiological functions of surrounding cells	
Endothelial cells	Endothelial cell senescence leads to impaired vasodilation and vascular dysfunction	Kohn et al. (2015), Kuzuya et al. (2001), Zieman et al. (2005), Krouwer et al. (2012)
	Endothelial cell senescence leads to disruption of the barrier function	
Cardiac fibroblast	Aging of fibroblasts may exacerbate cardiac fibrosis	Schelbert et al. (2017)
Vascular smooth muscle cells	Shortening telomeres	Chen et al. (2022)
	Increase the expression of p16 and p21,SA-β-gal activity and DNA damage	
	Promote the recruitment of monocytes, macrophages, and lymphocytes	
	Reducing the expression of SIRT6	
Valvular interstitial cells	Causing valve dysfunction	Yang et al. (2018)
	Increase ROS and form calcified nodules	

### 4.1 Cardiomyocytes

#### 4.1.1 Characteristics of cardiomyocyte senescence

**FIGURE 2:**
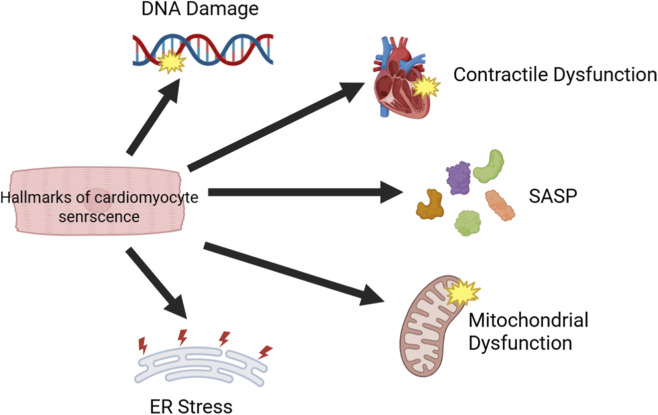
Characteristics of cardiomyocyte senescence. It mainly includes mitochondrial dysfunction and energy metabolism imbalance, telomere shortening and DNA damage, systolic dysfunction, ER stress and senescence secretion-related phenotypes.

Cardiomyocytes are the main cells of the heart, accounting for 80% of the total volume of heart cells and 30%∼40% of the total number of cells. Therefore, cardiomyocyte senescence can greatly affect heart function, leading to the development of age-related CVD. Since cardiomyocytes are terminally differentiated cells, the “irreversible cell cycle arrest” used as a definition of cellular senescence mentioned above does not apply here. However, when cardiomyocytes are exposed to a variety of stimuli, they also exhibit other senescence-associated features—phenomena that are consistent with the initiation of a form of senescence (Sweeney et al., 2023). We propose that cardiomyocyte senescence can be defined as a cellular process characterized by features of cellular senescence other than cell cycle arrest (Tang et al., 2020). As shown in Figure 2, It mainly includes mitochondrial dysfunction and energy metabolism imbalance, telomere shortening and DNA damage, systolic dysfunction, ER stress and senescence secretion-related phenotypes.

Mitochondrial dysfunction and imbalances in energy metabolism are associated with increased ROS production, which in turn leads to increased oxidative damage, including sulfhydryl oxidation, lipid peroxidation, and mitochondrial DNA (mtDNA) mutations. In addition to this, the imbalance towards hyperfusion of mitochondrial fission and fusion processes observed during cellular senescence leads to dysfunctional mitochondrial retention and accumulation of oxidized proteins, which may exacerbate cellular senescence.

In senescent cardiomyocytes, cellular and mitochondrial ROS accumulate and induce DNA damage and repair responses. Telomere shortening is the most common feature of DNA damage in senescent cells. Oxidative stress is a contributing factor to telomere shortening, which leads to activation of DNA damage signaling (Sharifi-Sanjani et al., 2017). Animal and human evidence suggests that post-mitotic cardiomyocyte senescence is mediated by length-independent telomere damage (Anderson et al., 2019). Importantly, telomere-specific DNA damage drives senescence-like phenotypes in cardiomyocytes. Therefore, DNA damage is an important driver and marker of cardiomyocyte aging.

Meanwhile, in senescent cardiomyocytes, systolic dysfunction is regulated by DNA and NAD depletion (Zhang et al., 2019), which has a causal relationship with superoxide enhancement (Yang et al., 2006). In senescent cardiomyocytes with impaired contractility, ER stress (ERS) is cumulative (Tang et al., 2020). ERS refers to a stress response in cells due to the accumulation of misfolded or unfolded proteins within the endoplasmic reticulum. ERS causes apoptosis and hypertrophy of cardiomyocytes (Xie et al., 2017; Zeng et al., 2020).

In addition, senescent cardiomyocytes also exhibit alterations in secretory phenotype. Anderson et al. recently identified a senescent phenotype in aged mouse cardiomyocytes, featuring the expression of cyclin-dependent kinase inhibitors (CDKIs) p16Ink4a and p21Cip1 and the induction of senescence-associated β-galactosidase (SA-β-gal) activity (Anderson et al., 2019; Ye et al., 2021). These cardiomyocytes also developed a secretory phenotype, which, albeit distinct from the classically defined SASP, contained multiple pro-fibrotic factors including TGF-β2, endothelin-3, and GDF15 (Anderson et al., 2019).

#### 4.1.2 Cardiomyocyte senescence regulatory network

**FIGURE 3:**
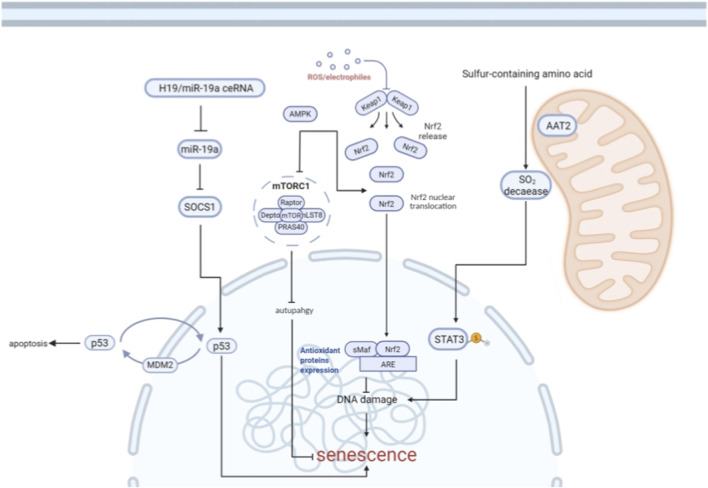
Regulatory network of cardiomyocyte senescence. This schematic illustrates the molecular mechanisms involved in cardiomyocyte senescence. The H19/miR-19a ceRNA axis inhibits miR-19a, leading to upregulation of SOCS1, which subsequently activates p53-mediated senescence pathways including CCF (cytoplasmic chromatin fragments), SASP (senescence-associated secretory phenotype), and apoptosis. mTORC1, regulated by AMPK and influenced by SOCS1, suppresses autophagy and contributes to DNA damage accumulation. ROS/electrophiles trigger the release and nuclear translocation of Nrf2, enhancing antioxidant protein expression via sMaf/ARE interaction, and counteracting oxidative stress. Mitochondrial metabolism of sulfur-containing amino acids via AAT2 and SO_2_ decaease activates STAT3 signaling, further promoting senescence. The interplay among these pathways orchestrates the balance between cell survival and senescence in cardiomyocytes.

As shown in Figure 3, cardiomyocyte senescence is a complex biological process that involves the regulation of multiple signaling pathways, as follows:

##### 4.1.2.1 mTOR signaling pathway

mTOR (mammalian target of rapamycin) is an important regulator of cell growth, metabolism and senescence, mainly through two complexes, mTORC1 and mTORC2 (Hua et al., 2019; Fu and Wu, 2023; Yang et al., 2022). The structure and function of mTORC1 and mTORC2 are conserved in eukaryotes. mTORC1 is mainly involved in protein synthesis and cell growth (Szwed et al., 2021). Its activity is regulated by nutrients (such as amino acids), energy status, and growth factors. When energy is sufficient, mTORC1 is not activated, promoting cell growth and metabolism; And in the state of energy deprivation or stress, mTORC1 is inhibited (Hua et al., 2019; Szwed et al., 2021). Clk-1 is a key protein that coordinates mitochondrial and nuclear activities. mTOR regulates Clk-1 through the AMPK-mTOR axis to modulate cellular respiration and aging (Zhang et al., 2017; López-Otín et al., 2013; Chandel, 2014; Sena and Chandel, 2012). Furthermore, as shown in Figure 3, mTORC1, which is regulated by AMPK and influenced by SOCS1, inhibits autophagy and promotes the accumulation of DNA damage. Therefore, inhibiting mTORC1 can enhance autophagy, leading to the clearance of unnecessary cytoplasmic proteins and the reduction of toxic metabolite accumulation, thereby alleviating cellular stress and prolonging lifespan (Marafie et al., 2024; Saxton and Sabatini, 2017). Moreover, the mTOR inhibitor rapamycin has been shown to contribute to lifespan extension in various organisms, making it the only known drug that directly regulates aging (Bjedov et al., 2010; Harrison et al., 2009; Powers et al., 2006; Robi et al., 2012); The main function of mTORC2 is the regulation of cytoskeletal tissue, and it is also involved in the regulation of cell survival and cell cycle progression. Its mechanism of action is relatively complex (Chong et al., 2010). Furthermore, mTORC2 may have the function of restoring metabolic homeostasis in response to nutrient fluctuations (Szwed et al., 2021).

Overactivation of mTOR may lead to cardiomyocyte hypertrophy and fibrosis, which in turn affects heart function. Therefore, moderate inhibition of the mTOR signaling pathway may have a protective effect on delaying the aging of cardiomyocytes (Huang et al., 2024). However, mTOR reduces the inflammatory response of cardiomyocytes and prevents cardiac insufficiency in cardiac hypertrophy (Song et al., 2010).

##### 4.1.2.2 AMPK signaling pathway

AMPK (adenylate-activated protein kinase) is an important kinase that regulates energy homeostasis and is a key protein involved in multiple signal transduction pathways (Ge et al., 2022). During energy stress, AMPK directly phosphorylates key factors involved in multiple pathways to restore energy balance. The effects of AMPK on metabolism can be broadly divided into two categories: inhibiting anabolism to minimize ATP consumption and stimulating catabolism to stimulate ATP production (Herzig and Shaw, 2018). AMPK activation can delay or block the aging process, which is of great significance in the treatment of cardiovascular diseases and other aging-related diseases (Ge et al., 2022). In cardiomyocytes, AMPK regulates aging through the following mechanisms:

Energy stress response: When cells are in a low-energy state, AMPK is activated, which in turn phosphorylates and activates TSC2, inhibiting the activity of mTORC1 (Gwinn et al., 2008). In addition, AMPK can also directly phosphorylate the Raptor subunit of mTORC1 and inhibit its function (Hawle et al., 2014). However, there is abundant evidence to support that inhibition of mTOR can counteract the characteristics of aging, including nutrient induction dysregulation, mitochondrial dysfunction, loss of protein homeostasis, etc. (Szwed et al., 2021; López-Otín et al., 2013).

Antioxidative stress: AMPK can activate Nrf2, promote the expression of antioxidant enzymes (such as HO-1 and GPX4), and alleviate the damage of oxidative stress to cardiomyocytes (Joo et al., 2016; Song et al., 2020).

Autophagy regulation: AMPK by inhibiting mTORC1, Protects the normal start of the autophagy process, which removes damaged mitochondria and protein aggregates within cells, thereby delaying cell senescence (Ge et al., 2022).

##### 4.1.2.3 Nrf2 signal passage

Nrf2 (nuclear factor E2-related factor 2) is a key regulator of cellular antioxidant response (Sajadimajd and Khazaei, 2018; He et al., 2020). Under normal conditions, Nrf2 binds to Kelch-like ECH-associated protein 1 (Keap1) and is degraded. When cells are subjected to oxidative stress, Nrf2 is released from Keap1 and transferred to the nucleus, activating the expression of a series of antioxidant genes (Chen and Maltagliati, 2018), reduces oxidative stress damage to cardiomyocytes. As shown in Figure 3, reactive oxygen species/electrophilic reagent triggers the release and nuclear translocation of Nrf2, which enhances the expression of antioxidant proteins through sMaf/ARE interaction, thereby counteracting oxidative stress.

##### 4.1.2.4 SO_2_/AAT_2_ aisle

Sulfur dioxide (SO2) has been considered as a new endogenous gas signal molecule after the discovery of nitric oxide (NO), carbon monoxide (CO) and hydrogen sulfide (H2S) (Huang et al., 2022; Kajimura et al., 2010; Yong et al., 2011). Endogenous SO_2_ in mammals is mainly produced by the metabolism of sulfur-containing amino acids, and aspartate aminotransferase (AAT) plays an important role in its production (Huang et al., 2022; Huang Y. et al., 2021). There are two isoforms of AAT, AAT_1_ is predominantly located in the cytoplasm and AAT_2_ is predominantly located in the mitochondrial matrix. It has been previously reported that AAT_2_ is a key enzyme in SO_2_ production, and its expression and activity directly affect the level of SO_2_ in cells. In cardiomyocytes, the SO_2_/AAT_2_ pathway can inhibit DNA damage and cellular senescence (Zhang S. et al., 2024).

##### 4.1.2.5 H19 RNA inhibits the miR-19a/socs1/p53 axis to regulate aging

Cardiomyocyte senescence is also regulated by non-coding RNA: Lnc-RNA-H19, as competing endogenous RNA (ceRNA) targeting the miR-19a/SOCS1/p53/p21 pathway, activates the p53/p21 pathway by inhibiting miR-19a up-regulating SOCS1 expression, thereby promoting cardiomyocyte senescence (Zhuang et al., 2021) as shown in Figure 3.

#### 4.1.3 Mechanism of cardiomyocyte senescence leading to age-related CVD

Impaired contractility and abnormal conduction patterns of senescent cardiomyocytes can lead to cardiomyopathy (Gude et al., 2018). For example, in a model of Duchenne muscular dystrophy, cardiomyocytes from mice lacking dystrophin exhibited a senescent phenotype (Chang et al., 2016). Senescence of cardiomyocytes may also increase the risk of ventricular arrhythmias (Chadda et al., 2018). Increased ROS in ventricular cardiomyocytes in senescent rats, which impairs their ability to couple with electrical pacing, suggests an increased risk of arrhythmias (Masoud et al., 2019). Senescent cardiomyocytes also maintain chronic inflammation by activating inflammatory signaling pathways, such as the Toll-like receptor 9-TLR9 pathway, and releasing pro-inflammatory cytokines. This inflammatory response not only affects the function of cardiomyocytes, but also promotes the occurrence of cardiovascular diseases such as atherosclerosis and myocardial fibrosis (Chen et al., 2022). In addition, senescent cardiomyocytes can also affect the physiological functions of surrounding cells by secreting SASP factors, resulting in the deterioration of the cardiac microenvironment. This alteration of cell-to-cell communication can further exacerbate cardiac dysfunction (He et al., 2024).

### 4.2 Endothelial cells

#### 4.2.1 Endothelial cell senescence characteristics

Endothelial cells participate in the formation of the vascular endothelium, regulating vascular dilation and vascular tone by secreting vasoactive compounds and growth factors (Colliva et al., 2020), playing a significant physiological role in maintaining vascular homeostasis, sustaining blood flow, participating in inflammatory responses, and promoting angiogenesis (Trimm and Red-Horse, 2023; Sturtzel, 2017). Studies have shown that endothelial cells account for more than 60% of non-cardiac cells in adult mice (Pinto et al., 2016), which can be observed in atherosclerotic plaques, failing hearts (especially those with diastolic dysfunction), and hearts with atrial fibrillation.

Unlike cardiomyocytes, endothelial cells have a strong ability to perform mitosis, and in the physiological state, the renewal of endothelial cells is mainly completed through mitosis (Bloom et al., 2023). Therefore, its senescence characteristics are similar to those of ordinary cells, including the upregulation of cell cycle inhibitors, the increase of senescence-related β-galactosidase activity, and the increase of SASP factor secretion (Bloom et al., 2023; Campisi and d'Adda di Fagagna, 2007). Morphologically, the cells become flattened, enlarged, arranged irregularly under laminar flow shear stress, and have increased adhesion to the basement membrane, preventing them from aligning correctly in the direction of blood flow. Functionally, endothelial cell senescence leads to reduce NO production and impaired barrier function (Bloom et al., 2023; Matsushita et al., 2001).

#### 4.2.2 Regulatory network of endothelial cell senescence

**FIGURE 4:**
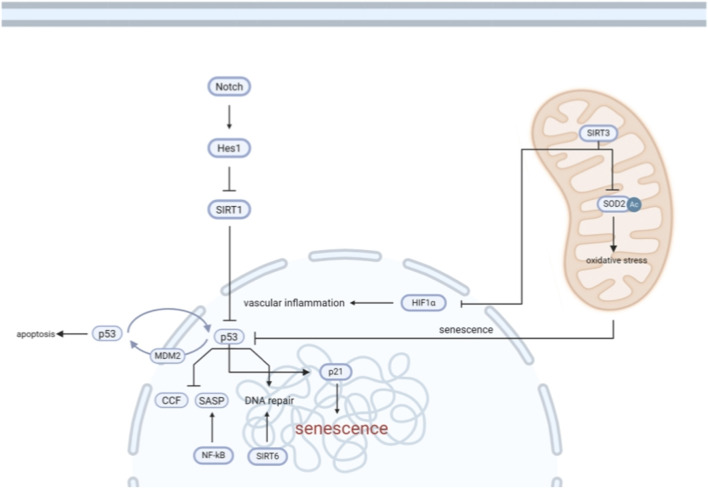
Regulatory pathways Involved in endothelial cell senescence. This diagram depicts the molecular signaling networks that regulate endothelial cell senescence. The Notch-Hes1 axis suppresses SIRT1 expression, which in turn leads to activation of the p53 pathway. Activated p53 promotes endothelial senescence through pathways involving p21, SASP (senescence-associated secretory phenotype), CCF (cytoplasmic chromatin fragments), and inhibition of DNA repair. SIRT6 supports DNA repair and limits NF-κB-mediated SASP expression. Concurrently, mitochondrial SIRT3 deacetylates and activates SOD2 to reduce oxidative stress; its suppression leads to increased mitochondrial ROS, contributing to cellular senescence. HIF1α further promotes senescence and vascular inflammation, highlighting the crosstalk between oxidative stress, mitochondrial dysfunction, and nuclear signaling in endothelial aging.

As shown in Figure 4, VEGF, KLF2, eNOS, NF-κB, and Sirtuins are key factors in the regulatory network of endothelial cell aging, and they play an important role in maintaining endothelial cell function and coping with aging-related changes. The following are the mechanisms by which these factors work in the regulation of endothelial cell aging:

##### 4.2.2.1 VEGF (vascular endothelial growth factor)

VEGF is a key factor in maintaining endothelial cell function and vascular homeostasis. There are three types of VEGF receptors, including VEGFR-1, VEGFR-2, and VEGFR-3 (Yamazaki and Morita, 2006). VEGF regulates endothelial cell proliferation, migration, and survival through VEGFR2 (KDR) (Melincovici et al., 2018). In senescent endothelial cells, the function of the VEGF signaling pathway is impaired, which is manifested by the downregulation of KDR expression. Through genetic reprogramming, such as overexpression of KDR in p16^Ink4a^ + senescent endothelial cells, its proliferative ability can be restored and pathological states such as liver fibrosis can be alleviated (Zhao et al., 2024).

##### 4.2.2.2 KLF2 (Krüppel-like factor 2)

KLF2 is a transcription factor that maintains endothelial cell homeostasis primarily by regulating endothelial cell antioxidant enzymes and inhibiting endothelial cell activation by various pro-inflammatory stimuli (Li et al., 2021; Atkins and Jain, 2007). KLF2 upregulates the expression of eNOS (Atkins and Jain, 2007), thereby promoting the production of nitric oxide (NO), a key regulator of endothelial cell function. During aging, the activity of KLF2 may be inhibited (Gao et al., 2019; Zhang et al., 2023), resulting in decreased antioxidant capacity and an enhanced inflammatory response in endothelial cells.

##### 4.2.2.3 eNOS (endothelial nitric oxide synthase)

eNOS is a key enzyme for NO production in endothelial cells (Takahashi and Mendelsohn, 2003; Daiber et al., 2019), NO has a variety of functions such as vasodilation, anti-inflammatory, and antioxidant. In addition, there is evidence that NO may be a key vasodilator molecule in the prevention of cellular aging (Hayashi et al., 2006; Hayashi et al., 2008). The decrease in eNOS activity in senescent endothelial cells leads to a decrease in NO production, which in turn leads to endothelial dysfunction (Matsushita et al., 2001).

##### 4.2.2.4 NF-κB (nuclear factor Kappa B)

NF-κB is a key transcription factor in the regulation of inflammation and cellular senescence (Pisani et al., 2022; Khongthong et al., 2019; Lee et al., 2021). During endothelial cell senescence, NF-κB is activated, resulting in increased expression of senescence-associated secretory phenotype (SASP) factors (Calubag et al., 2024). These factors not only exacerbate endothelial cell senescence, but may also affect surrounding cells through paracrine effects. Inhibition of NF-κB activity can alleviate endothelial cell senescence and inflammatory responses (Bloom et al., 2023).

##### 4.2.2.5 Sirtuins

Sirtuins (SIRTs) are NAD^+^-dependent deacetylases and ADP-ribosyltransferases (Houtkooper et al., 2012). When the cell When NAD^+^ is elevated, SIRT1 and SIRT6 promote cellular repair and stress resistance by mediating tumor suppressors and DNA damage repair pathways. As a result, SIRTs have become important mediators of endothelial cell senescence (Houtkooper et al., 2012). Inhibition of SIRT1 and SIRT6 increases senescence of endothelial cells in culture. Inhibition of SIRT1 in endothelial cells increases p53 activation, which may explain how SIRT1 mediates endothelial cell senescence. In contrast, the mechanism of SIRT6 reduction leading to senescence appears to be upstream of p53-p21 activation and is related to SIRT6’s role in telomere protection and DNA repair (Bloom et al., 2023). As shown in Figure 4, SIRT6 supports DNA repair and restricts NF-κB-mediated SASP expression.

Furthermore, SIRT3 is a key regulator of antioxidant and metabolic function. This molecule localizes to mitochondria and protects cells from oxidative stress. As shown in Figure 4, mitochondrial SIRT3 alleviates oxidative stress by deacetylation and activation of SOD2; its inhibition leads to an increase in mitochondrial reactive oxygen species (ROS), thereby promoting cellular senescence. Elevated SIRT3 expression reduces endothelial cell senescence in culture (Chen T. et al., 2021). In contrast, senescent endothelial cells decreased SIRT3 levels. Systemic overexpression of SIRT3 protects mice from angiotensin 2-induced endothelial dysfunction and shortens aortic senescence (Dikalova et al., 2020). These findings imply an important role for SIRT3 and mitochondrial function in endothelial cell senescence.

#### 4.2.3 Mechanism of endothelial cell senescence leading to age-related CVD

Endothelial cell senescence leads to impaired vasodilation and vascular dysfunction, leading to conditions such as atherosclerosis, heart failure with preserved ejection fraction (HFpEF), or pulmonary hypertension. For example, vascular endothelial cell dysfunction has been shown to affect arteriosclerosis through cell adhesion molecules (CAMs), MMPs, and collagen molecules (COLs) (Kohn et al., 2015; Kuzuya et al., 2001; Zieman et al., 2005). Furthermore, senescent endothelial cells produce pro-inflammatory secreted associated proteins (SASP), including TNF-α, IL-1, IL-6, and IL-8 (Prattichizzo et al., 2018; Matacchione et al., 2021). These cytokines, coupled with increased expression of vascular adhesion molecules on the lumen surface, enhance the recruitment of circulating immune cells to the vascular wall (Matacchione et al., 2021). Macrophage infiltration into the intima and their phagocytosis of intimal cholesterol to form foam cells represent a critical early stage in atherosclerotic plaque development. The presence of senescent cells in endothelial cells is likely to promote or accelerate this process (Tabas et al., 2016).

The destruction of the barrier function is also a major reason. As the inner layer of blood vessels, the endothelium forms a selective osmotic barrier that regulates the passage of molecules between the blood and peripheral cells and tissues. The permeability of the endothelium is tissue-specific and mediated by connexins (Claesson-Welsh et al., 2021). Studies have shown that culturing endothelial cells until they age results in the disintegration of the connexin cadherin-5 (also known as vascular endothelial cadherin) and the tight junction protein ZO-1, as well as a decrease in the connexins occludin and claudin-5 (Krouwer et al., 2012). In addition, non-senescent cells exhibit dysfunctional tight junctions when co-cultured with senescent endothelial cells (Krouwer et al., 2012). Therefore, endothelial cell senescence will lead to the destruction of barrier function, which in turn leads to a variety of diseases.

### 4.3 Comparison of regulatory networks of cardiomyocyte and endothelial cell senescence

Although cardiomyocytes (post-mitotic) and endothelial cells (mitotically competent) exhibit distinct senescence triggers and phenotypes, their regulatory networks converge on several key hubs. For instance, p53 activation serves as a common effector in both cell types, albeit initiated via different upstream signals (e.g., DNA damage in cardiomyocytes vs. SIRT1 inhibition in endothelial cells). Oxidative stress is another universal driver, while the response diverges at the level of specific mediators—cardiomyocytes heavily rely on the H19/miR-19a/SOCS1 axis and SO2/AAT2 pathway, whereas endothelial senescence is prominently regulated by KLF2/eNOS/NO axis and Sirtuin family proteins. This comparison underscores both shared and cell-type-specific vulnerabilities that could be exploited for targeted therapies.

### 4.4 Miscellaneous

In addition to cardiomyocytes and endothelial cells, the senescence of fibroblasts, vascular smooth muscle cells, and valvular stromal cells is associated with age-related cardiovascular disease (Bloom et al., 2023). For example, senescent vascular smooth muscle cells can lead to the development of atherosclerosis (Wang and Bennett, 2012); Aging of valve stromal cells can lead to valve dysfunction (Yang et al., 2018); Cardiac fibrosis may be exacerbated by the aging of fibroblasts in chronic disease (Schelbert et al., 2017).

## 5 Challenges and outlook

Although current research on age-related cardiovascular disease has made some progress, it still faces many challenges in in-depth mechanism analysis and clinical application transformation, mainly including the following three aspects: mechanism complexity, model limitations and clinical transformation bottlenecks. Firstly, cellular senescence involves the cross-regulation of multiple signaling pathways, and different types of cells have their own unique mechanisms of cellular senescence. It is difficult to fully elucidate the specific mechanisms in different cardiovascular cell types. Secondly, some of the conclusions presented in this paper are obtained in animal models or *in vitro* cultures, but the existing animal models are difficult to fully simulate the chronic progression characteristics of human age-related CVD, and the *in vitro* culture system cannot reproduce the dynamic interaction of the *in vivo* microenvironment, so the model has certain limitations. Lastly, although some drugs have been shown to be effective in age-related CVD, their long-term safety, targeting specificity, and impact on non-senescent cells still need to be verified.

These challenges highlight the need for continued exploration into the role of cellular senescence in age-related cardiovascular diseases. Although we have summarized some of the mechanisms by which cellular senescence leads to age-related CVD, this is not sufficient. Furthermore, by targeting the regulatory networks and mechanisms of cellular senescence—particularly governing SASP—we can develop targeted strategies to either eliminate senescent cells or slow the aging process, thereby addressing age-related cardiovascular disease (CVD). As shown in Table 2, these approaches also hold promise for clinical translation.

**TABLE 2:** Cardiovascular drugs targeting the regulatory networks and mechanisms of cellular senescence.

Methods	Agents	Primary mechanism of action	Effect	Clinical proof
Senomorphics	Dapagliflozin	Activates the SIRT1 signaling pathway and suppresses downstream pro-inflammatory and pro-oxidative pathways, such as NF-κB (Tai et al., 2023)	Attenuates endothelial cell senescence	NCT05975528 (still recruiting)
	Resveratrol	Activates SIRT1 and AMPK (Un et al., 2009)	Attenuates endothelial cell senescence	NCT01842399
	Rapamycin	mTOR inhibition; SASP suppression (Selvarani et al., 2021)	Attenuates vascular and endothelial senescence	NCT01649960
	Metformin	AMPK activation; NF-κB inhibition (Kulkarni et al., 2020)	Suppresses SASP and improves endothelial function	—
Senescent cell killing	Dasatinib + Quercetin	Dasatinib inhibits the JAK/STAT signaling axis, thereby inducing apoptosis of senescent cells. Quercetin activates AMPK and the Nrf2 pathway while suppressing MAPK/ERK signaling. When used in combination, dasatinib and quercetin synergistically modulate the PI3K/Akt pathway as well as the mTOR and p53 signaling axes, leading to enhanced elimination of senescent cells (Nieto et al., 2024)	Attenuates the anti-apoptotic resistance of senescent cells and improves cardiovascular function (Nieto et al., 2024; Xuan and Duan, 2024)	NCT04994561

Beyond these pharmacological agents that target senescent cells or their secretome, future management of age-related CVD may involve more proactive and personalized strategies. We have demonstrated that cellular senescence and age-related CVD are closely related, and whether a risk assessment system can be established based on aging biomarkers (such as telomere length and γH2AX level) to achieve early warning and hierarchical management of age-related CVD is an important direction for future research. Finally, the integration of cutting-edge technologies—including nanotechnology, gene editing, and artificial intelligence (AI)—offers unprecedented opportunities to improve the diagnosis, risk stratification, and treatment of age-related cardiovascular disease (CVD) (Zhu et al., 2025; Chen W. et al., 2023; Chen S. et al., 2021; Chen S. et al., 2023; Ouyang et al., 2023). Nanotechnology-based delivery systems enable tissue-targeted and cell-specific modulation of senescence-associated pathways, potentially reducing off-target toxicity and improving therapeutic efficacy (Li et al., 2023; Fadaka et al., 2021; Alimardani et al., 2023; Xu et al., 2021). Gene editing technologies, such as CRISPR/Cas systems, provide powerful tools to interrogate and correct causal molecular drivers of cardiovascular aging, while AI-driven algorithms facilitate the integration of high-dimensional data derived from imaging, omics, and clinical records to enhance early detection and personalized risk prediction (Pacesa et al., 2024; Huang S. et al., 2021). Together, these approaches hold significant promise to translate aging biology into clinically actionable strategies.

Despite this potential, the implementation of aging biomarker–based risk assessment systems faces several practical and conceptual challenges. The cost and technical complexity of multi-omics profiling and longitudinal biomarker monitoring remain substantial barriers, particularly in large-scale population screening. Besides, although numerous biomarkers correlate with age-related CVD risk, establishing clear causal relationships—rather than associative thresholds—remains difficult, complicating their clinical interpretation and regulatory acceptance. Additionally, the lack of standardized cutoff values, inter-platform variability, and limited validation across diverse populations further restrict clinical translation. Addressing these challenges will require coordinated efforts to refine biomarker selection, validate causality through functional and interventional studies, and leverage AI-based modeling to integrate heterogeneous data into robust, clinically meaningful risk assessment frameworks.

## 6 Conclusion

Cellular senescence is an irreversible state of cell cycle arrest, characterized by DDR activation, telomere shortening, SASP, and metabolic dysfunction. These features are closely related to the aging of the cardiovascular system, leading to functional deterioration of key cell types such as cardiomyocytes, endothelial cells, etc. Age-related CVD (e.g., atherosclerosis, heart failure, hypertension, and arrhythmias) refers to cardiovascular diseases that are closely associated with increasing age, with cellular senescence, chronic inflammation, and tissue damage. Its pathogenesis is closely related to cellular senescence. We focus on how the senescence of cells affects SASP, how SASP changes the senescence from a cellular autonomous phenomenon to a systemic problem, the regulatory network of senescence of cardiomyocytes and endothelial cells (such as mTOR, AMPK, Nrf2, Sirtuins and so on) and the mechanism of senescence of CVD. Finally, we recognize that the current research faces major challenges such as mechanistic complexity, model limitations, and bottlenecks in clinical translation, and provide an outlook for future research.
